# Characterization of the L-form switch in the Gram-negative pathogen *Streptobacillus moniliformis*

**DOI:** 10.1093/femsle/fnab156

**Published:** 2021-12-15

**Authors:** David M Roberts, Jeff Errington, Yoshikazu Kawai

**Affiliations:** Centre for Bacterial Cell Biology, Biosciences Institute, Medical School, Newcastle University, Baddiley-Clark Building, Richardson Road, Newcastle upon Tyne NE2 4AX, UK; Centre for Bacterial Cell Biology, Biosciences Institute, Medical School, Newcastle University, Baddiley-Clark Building, Richardson Road, Newcastle upon Tyne NE2 4AX, UK; Centre for Bacterial Cell Biology, Biosciences Institute, Medical School, Newcastle University, Baddiley-Clark Building, Richardson Road, Newcastle upon Tyne NE2 4AX, UK

**Keywords:** L-form bacteria, *Streptobacillus moniliformis*, cell wall, outer membrane, antibiotics

## Abstract

Almost all major classes of bacteria are surrounded by a peptidoglycan cell wall, which is a crucial target for antibiotics. It is now understood that many bacteria can tolerate loss of the cell wall provided that they are in an isotonic environment. Furthermore, in some cases the cells can continue to proliferate in a state known as the L-form. L-form proliferation occurs by an unusual blebbing or tubulation mechanism that is completely independent of the normally essential division machine or cell wall synthetic enzymes, and is resistant to cell wall-active antibiotics. However, the growth is limited by reactive oxygen species generated by the respiratory chain pathway. In this work, we examined the walled to L-form transition in a pathogenic Gram-negative bacterium, *Streptobacillus moniliformis*, which naturally lacks the respiratory chain pathway, under aerobic conditions. L-form-like cells often emerged spontaneously, but proliferation was not observed unless the cells were treated with cell wall-active antibiotics. Time-lapse imaging revealed that cell division of *S. moniliformis* L-forms involves unusual membrane dynamics with an apparent imbalance between outer membrane and cytoplasmic volume growth. The results suggest that outer membrane expansion may be an important general factor for L-form proliferation of diderm bacteria.

## Teaser

This work investigates general factors involved in a process of the walled to L-form switching using a Gram-negative bacterium, *Streptobacillus moniliformis*, which was the first organism in which the L-form state was reported.

## INTRODUCTION

The peptidoglycan (PG) cell wall is widely conserved across the bacterial domain and normally essential for viability. It is a crucial determinant of bacterial cell shape and an elastic structure that counteracts cell turgor. PG is also an important target for antibiotics, especially β-lactams, as well as a source of trigger molecules for innate immune responses. A few unusual groups of bacteria lack a cell wall, including pathogenic *Mycoplasma* (Errington *et al*. 2016; Errington 2017). It is also well known that treatment of many species of bacterial cells with cell wall-active antibiotics can convert them into a cell wall-free state called the L-form. The major physiological change that arises in L-forms is a requirement for protection from osmotic lysis, but importantly, this is fulfilled in host environments, including urine or blood (Dienes and Weinberger 1951; Allan, Hoischen and Gumpert 2009; Errington *et al*. 2016; Kawai, Mickiewicz and Errington 2018; Mickiewicz *et al*. 2019; Markova 2020). Freshly induced L-forms are genetically identical or nearly identical to their parental walled bacteria and often are able to switch back to the walled state (Kawai, Mercier and Errington 2014; Mickiewicz *et al*. 2019). L-forms have mainly been identified as antibiotic-resistant organisms in samples from humans (Domingue and Woody 1997; Allan, Hoischen and Gumpert 2009; Errington *et al*. 2016), and various reports have provided evidence for an important role in chronic or recurrent infections (Onwuamaegbu, Belcher and Soare 2005; Kawai, Mickiewicz and Errington 2018; Mickiewicz *et al*. 2019; Markova 2020).

The earliest report defining an L-form was published in 1935 by Emmy Klieneberger (Klieneberger 1935). She isolated a ‘pleuropneumonia-like’ organism, which she called L1, in a culture of the Gram-negative rod-shaped bacterium, *Streptobacillus moniliformis*, from the blood of rats. A few years later, she concluded that L1 and *S*. *moniliformis* were different forms of the same organism (Klieneberger 1949; Dienes and Weinberger 1951). Meanwhile, organisms similar to L1 of *S*. *moniliformis* were reported for many bacteria, often following treatment with penicillin, and they were also called ‘L-form’ bacteria (Dienes and Weinberger 1951). Although there is a large literature on L-forms, publications peaked around the 1970s and nothing was known about the molecular mechanisms underlying the generation of L-forms. However, in recent years researchers have revisited the L-form problem with modern molecular biology tools (Leaver *et al*. 2009; Mercier, Kawai and Errington 2013, 2014; Kawai *et al*. 2015, 2019; Studer *et al*. 2016; Ramijan *et al*. 2018; Wu *et al*. 2020; Chikada *et al*. 2021). This work has started to uncover central conserved properties of the unusual L-form state, as well as the genetic and other mechanisms involved in the process of L-form formation and proliferation. However, most of the work has been limited to experimentally tractable model bacteria under laboratory conditions.

L-form proliferation is independent of the normally essential division machine or cell wall synthetic enzymes in various bacteria (Leaver *et al*. 2009; Mercier, Kawai and Errington 2014; Studer *et al*. 2016). Instead, it seems to involve a simple biophysical effect based on an increased rate of cell surface growth relative to volume, driving cell shape deformations that lead to spontaneous scission (Mercier, Kawai and Errington 2013). However, because reactive oxygen species originating from the respiratory chain (RC) pathway are abnormally increased in cell wall-deficient cells, the reduction of RC activity, high levels of ROS scavengers or depletion of oxygen is normally required to support robust L-form growth in a Gram-positive *Bacillus subtilis* or a Gram-negative *Escherichia coli* (Dienes and Weinberger 1951; Huber and Brinkley 1977; Kawai *et al*. 2015, 2019; Chikada *et al*. 2021). Crucially, these effects do not occur in a Gram-positive bacterium, *Enterococcus faecium*, which lacks the RC pathway (Kawai *et al*. 2019).

*Streptobacillus* *moniliformis* is one of the two causes of rat-bite fever, which was first described in 1925 by Levaditi, Nicolau and Poincloux (1925) and is an acute febrile human illness with severe toxic symptoms, which can sometimes lead to complications such as arthritis, endocarditis and pneumonia. It is acquired primarily from rodents, especially rats (Elliott 2007). Biochemical tests showed negative reactions for oxidase and catalase (Elliott 2007). The type strain 9901 (collection number DSM 12112 in the Deutsche Sammlung von Mikroorganismen und Zellkulturen or ATCC 14647 in the American Type Culture Collection) was isolated from a patient with rat-bite fever, and recently its genome sequencing was completed as the second species from the phylum Fusobacteria (Nolan *et al*. 2009). The genome sequencing confirmed the lack of the RC pathway, except for adenosine triphosphate (ATP) synthase, in this organism. In this work, we reexamined the formation of L-forms by *S*. *moniliformis* type strain 9901 during antibiotic treatment under aerobic culture conditions and applied recently developed time-lapse methods to characterize the process of L-form generation. We visualized the emergence of L-forms from parental walled cells, and their growth and scission events, which were associated with unusual membrane dynamics, even under aerobic conditions. Our results also suggest that outer membrane (OM) expansion is a critical factor for proliferation of *S. moniliformis* L-forms.

## MATERIALS AND METHODS

### Strain and growth conditions

*Streptobacillus moniliformis* type strain 9901 (DSM 12112, ATCC 14647) is used in this work (Levaditi, Nicolau and Poincloux 1925). Nutrient broth or agar (NB or NA; Oxoid, Hampshire, UK) containing 20% calf serum was used for the growth at 30 or 37°C under aerobic conditions. Isotonic sucrose medium, which is composed of 2× magnesium–sucrose–maleic acid (MSM), pH 7 (40 mM magnesium chloride, 1 M sucrose and 40 mM maleic acid), mixed 1:1 with 2× NB or NA, was used as a standard condition for L-form growth as described previously (Mercier, Kawai and Errington 2014; Kawai *et al*. 2019). One molar sucrose in 2× MSM was replaced by 1 M glucose, succinate, NaCl or sorbitol as necessary. Antibiotic phosphomycin or ampicillin (200 µg/ml) was added when required.

### Microscopy and image analysis

For snapshot live-cell imaging, walled cells were mounted on microscope slides covered with a thin film of 1.2% agarose in water, essentially as described previously (Glaser *et al*. 1997). L-forms were mounted on microscope slides without agarose.

All microscopy experiments were conducted using a Nikon Ti microscope equipped with a Nikon CFI Plan Apo DM Lambda 100× oil objective and a Photometrics Prime camera, using MetaMorph software (version 7.7, Molecular Devices, CA USA). Images and movies were analysed and processed using Fiji (https://imagej.net/Fiji) (Schindelin *et al*. 2012).

For time-lapse imaging, cells were placed in Gene Frames (Thermo Fisher, MA, USA) on NA containing serum with 200 μg/ml ampicillin. Time-lapse microscopy in liquid conditions was conducted using the CellASIC ONIX microfluidic system (Merck Millipore, MA, USA). B04A ONIX plates for bacteria (Merck Millipore, MA, USA) were loaded with 300 μl medium (NB with serum) containing appropriate supplements when required (MSM, 200 μg/ml ampicillin; see figures for details) and incubated at 30°C for 20 min. The imaging chamber was prewashed with media at 5 psi for 10 min before adding the cells. One hundred microliter cells taken from liquid batch cultures were then loaded into the imaging chambers at 4 psi for 30 s, prior to a channel washing step for a further 30 s at 3 psi. During the time lapse, media perfusion was maintained at 2 psi and the temperature at 32°C, with images acquired every 10 min. All flow rate and media switching steps were controlled using the CellASIC ONIX FG software (v 5.5.1.0).

To measure changes in the area (i.e. growth) of the OM, L-forms (or L-form-like cells) were selected from time-lapse experiments using the CellASIC ONIX system (Merck Millipore, MA, USA), after treatment with ampicillin (Video S4, Supporting Information) or sucrose (Video S2, Supporting Information). In all cases, cells were selected from the same trap height in the ONIX device. Only structures that could be clearly identified as individual cells (i.e. no overlap with any other structures), and that were clearly L-form-like, were selected. The OM can be detected as a phase light outline. Using the segmented line function in Fiji (https://imagej.net/Fiji) (National Institutes of Health (NIH)), a line was manually drawn around the OM for each frame over a 90 min period, and the area measured and extracted for each cell.

## RESULTS AND DISCUSSION

### Spontaneous formation of L-form-like structures in *S. moniliformis*

**Figure 1.**
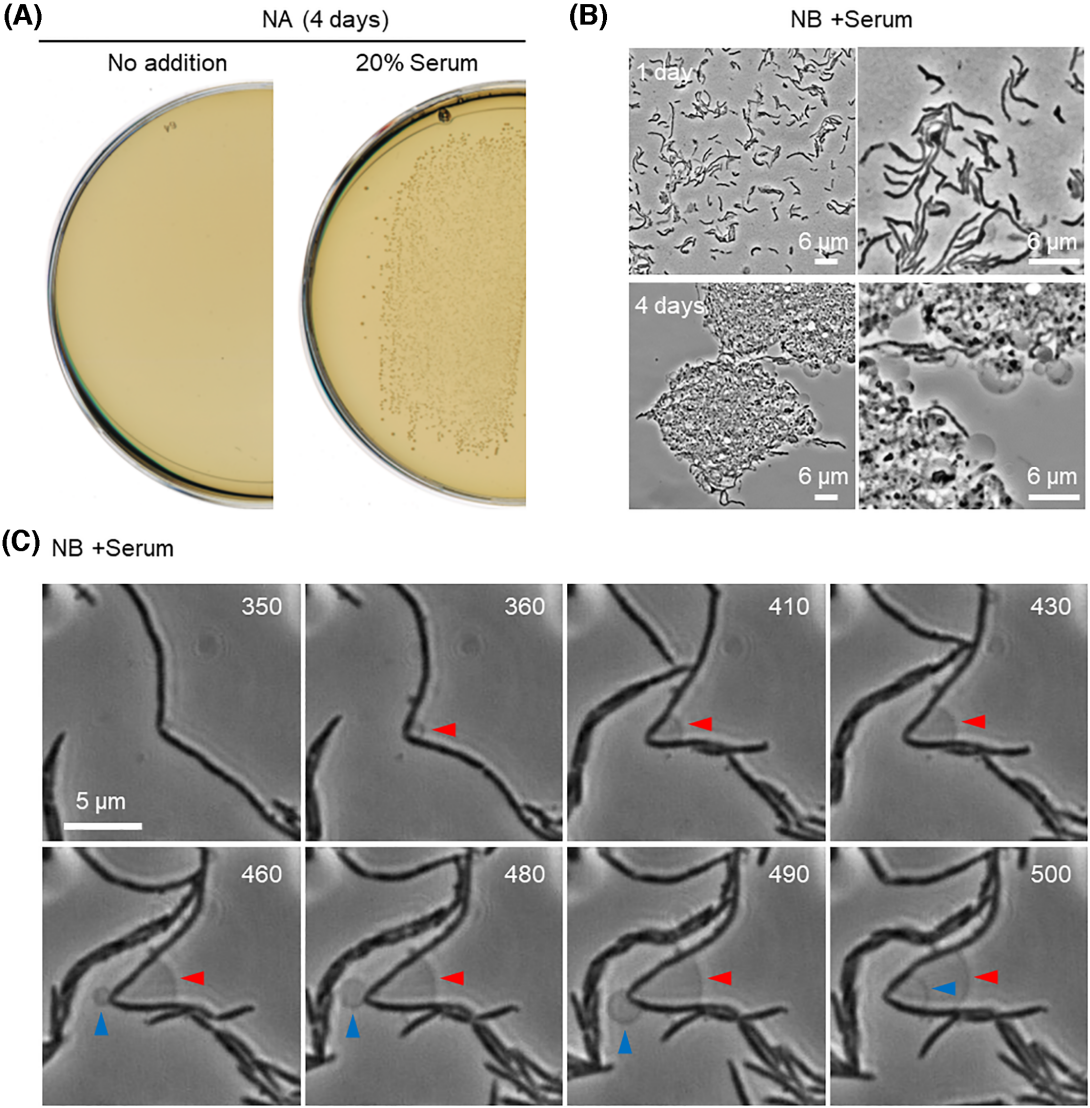
Growth and morphology of *S. moniliformis*. **(A)** Growth of *S. moniliformis* type strain 9901 on NA plate containing 20% calf serum at 30°C. **(B)** Phase-contrast micrographs of *S. moniliformis* in NB with serum after 1 or 4 days of incubation at 30°C. **(C)***Streptobacillus moniliformis* cells were cultured in NB with serum in the CellASIC ONIX microfluidic system for time-lapse microscopy. Individual frames are extracted from Video S1 (Supporting Information). Numbers in the top right corner of each frame represent time (min) elapsed in the video. Arrowheads represent spontaneous formation of L-form-like structures.

*Streptobacillus moniliformis* type strain 9901 was aerobically cultured on NA plates at 30°C, with or without 20% serum (Fig. 1A). Colonies started to appear after 2–3 days of incubation, but only in the presence of serum, as previously indicated (Dienes and Weinberger 1951; Elliott 2007). A few colonies were suspended in liquid NB supplemented with serum and incubated overnight. Microscopic observation of the culture revealed the typical cell morphologies previously described (Elliott 2007; Nolan *et al*. 2009). They were pleomorphic, mainly filamentous or short rods, with a straight or slightly curved shape. A few cells also had lateral bulbar swellings or a phase pale appearance indicative of cell lysis, on agarose-coated microscope slides (Fig. 1B, upper panels). After further incubation (3–4 days), the cells formed tangled clumps containing L-form-like structures (Fig. 1B, lower panels). This is consistent with earlier reports that described spontaneous emergence of L-forms in *S. moniliformis* (Dienes and Weinberger 1951; Freundt 1956). To directly visualize this, we carried out time-lapse experiments in a microfluidic device. A representative video is shown in Video S1 (Supporting Information). Figure 1C shows an example of cropped individual frames from the video documenting the spontaneous emergence of L-form-like structures from a parental rod (arrowheads). However, no significant growth or division of the L-form-like structures was detected.

### Osmolytes facilitate the formation of L-form-like structures but not growth

**Figure 2.**
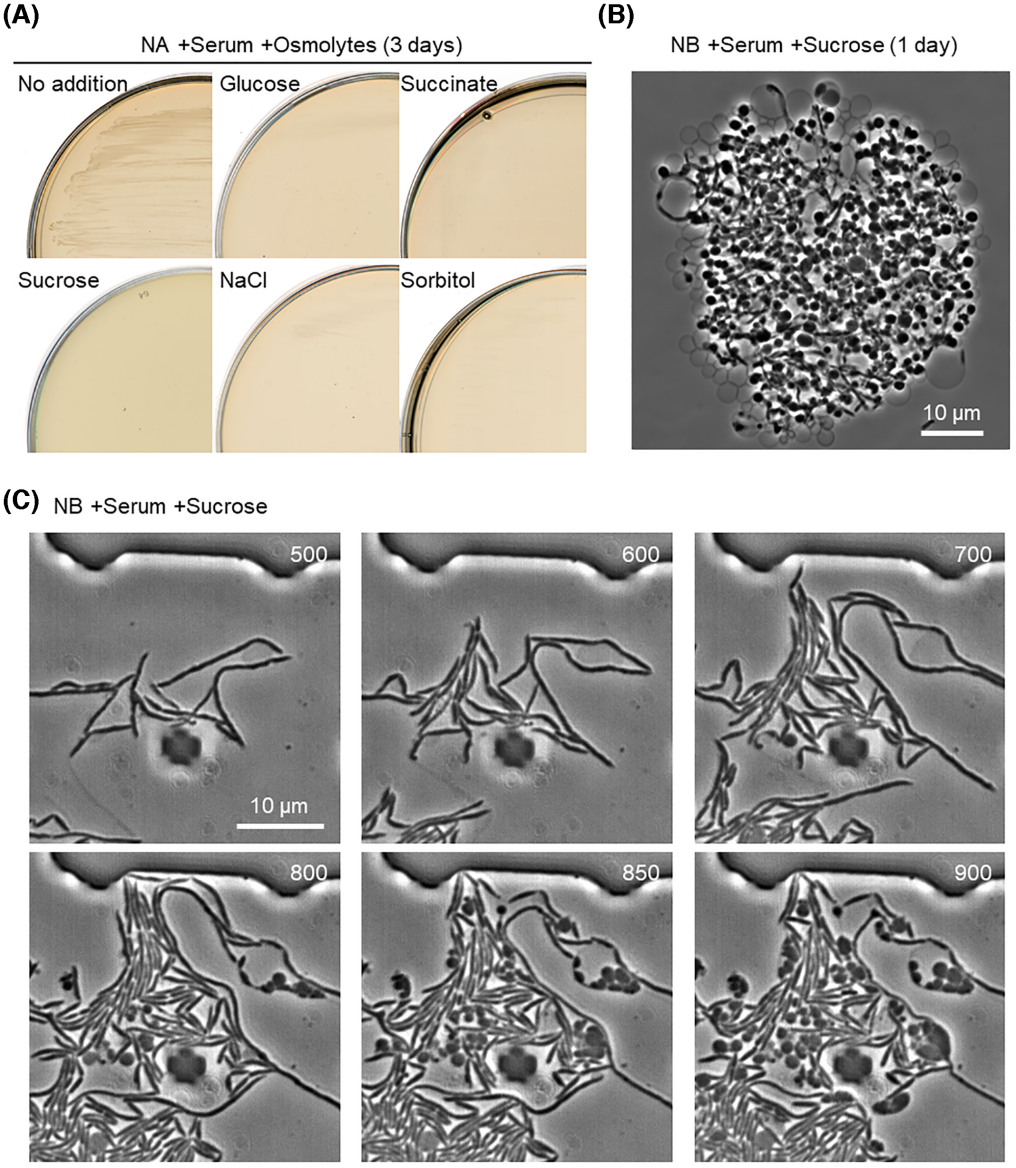
Effects of hypertonic solutions on growth and morphology of *S. moniliformis*. **(A)** Growth of *S. moniliformis* on NA plate containing serum supplemented with or without 0.5 M sucrose (MSM), glucose, succinate, NaCl or sorbitol at 30°C. **(B)** Phase-contrast micrograph of *S. moniliformis* in NB containing serum and MSM (0.5 M sucrose) at 30°C. **(C)***Streptobacillus moniliformis* cells were cultured in NB containing serum and MSM in the CellASIC ONIX microfluidic system for time-lapse microscopy. Individual frames are extracted from Video S2 (Supporting Information). Numbers in the top right corner of each frame represent time (min) elapsed in the video.

L-form growth in standard culture media, which are normally hypotonic, generally requires osmotic support to avoid cell lysis due to internal turgor pressure. Sucrose is often added to complex rich media to provide an isotonic environment, which can support L-form growth of a wide range of bacterial species, both Gram-positive and -negative (Mercier, Kawai and Errington 2014). In the presence of extra sucrose, however, *S. moniliformis* growth was severely impaired on NA plates containing serum (Fig. 2A, left panels). We tested several kinds of osmolytes but they also prevented growth (Fig. 2A). It seems that hypertonic solutions, which are normally required for L-form growth, act as an impediment to *S. moniliformis* growth. The growth defect due to extra sucrose was also seen in liquid media where the cells again formed tangled clumps containing abundant L-form-like structures even after overnight incubation (Fig. 2B). However, although time-lapse microscopy confirmed the induction of L-form-like structures after the addition of sucrose (Fig. 2C; Video S2, Supporting Information), the L-form cells did not show significant growth. This is similar to several species of filamentous actinomycetes that have a natural ability to extrude nonproliferative wall deficient cells when exposed to high levels of osmolytes (Ramijan *et al*. 2018).

### Cell wall-active antibiotics promote L-form growth

**Figure 3.**
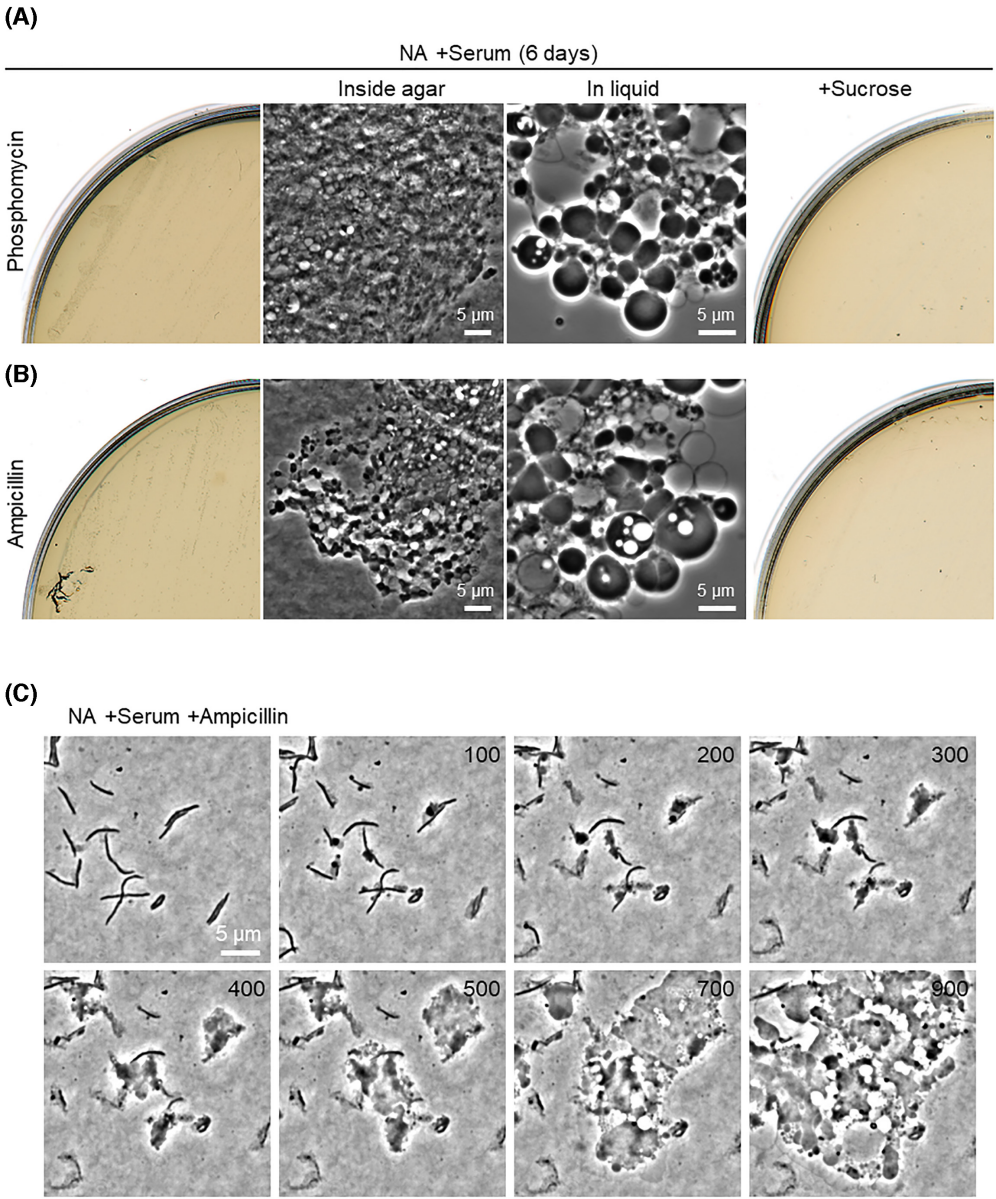
L-form generation in *S. moniliformis* by cell wall-active antibiotics. Growth of *S. moniliformis* on NA or NA/MSM (0.5 M sucrose) plates containing serum with or without 200 μg/ml phosphomycin **(A)** or ampicillin **(B)** at 30°C. Phase-contrast micrographs of *S. moniliformis* cells embedded in agar were taken from the plates (inside agar), or the cells were suspended in liquid NB (in liquid). **(C)***Streptobacillusmoniliformis* cells were placed in Gene Frames on NA containing serum with 200 μg/ml ampicillin. Individual frames are extracted from Video S3 (Supporting Information). Numbers in the top right corner of each frame represent time (min) elapsed in the video.

We have previously reported that L-form formation and proliferation can be efficiently induced by inhibiting *de novo* PG precursor synthesis in a wide range of bacteria under laboratory conditions (Mercier, Kawai and Errington 2014). Phosphomycin is a broad-spectrum bactericidal antibiotic that prevents the first committed step in the PG precursor pathway by inhibiting the MurA enzyme (UDP-*N*-acetylglucosamine enolpyruvyl transferase) (Silver 2017). We cultured *S. moniliformis* on NA plates with serum in the presence of phosphomycin. Detectable small colonies appeared after 4 days of incubation. *Streptobacillus moniliformis* colonies normally grow on the surface of agar plates, but these small colonies grew inside the agar and the cells seemed to form tangled clumps within the agar (Fig. 3A, inside agar). The preference of L-forms for growth inside the agar has been reported in several bacteria, including *E. coli* and *Staphylococcus aureus* (Glover *et al*. 2009; Han *et al*. 2014). We took the small colonies embedded in agar and suspended them in liquid medium. Microscopic observation of the cells revealed a typical L-form morphology (Fig 3A, in liquid). No L-form growth occurred in the presence of extra sucrose (Fig. 3A, right), as seen for normal walled cell growth in the absence of antibiotics (Fig. 2A). β-lactam antibiotics, which prevent the last cross-linking step of new PG insertion in the wall by inhibiting the transpeptidase activity of penicillin binding proteins (Lovering, Safadi and Strynadka 2012), have been used for L-form induction in *S. moniliformis* previously (Dienes and Weinberger 1951). Indeed, the β-lactam antibiotic ampicillin also worked to promote L-form growth, in a similar manner to phosphomycin (Fig. 3B). L-form growth induced by ampicillin was again inhibited in the presence of sucrose (Fig. 3B).

To directly visualize the L-form switch from parental rods, we incubated *S. moniliformis* cells on microscope slides coated with NA containing serum in the presence of ampicillin and captured images in a time-lapse series. A representative video is shown in Video S3 (Supporting Information). Figure 3C shows an example of cropped individual frames from the video. The growth of *S. moniliformis* was immediately arrested after ampicillin treatment, followed by bulging and deformation (∼100 min). The cells then grew and propagated in the classical manner described for L-forms, involving a range of unusual blebbing events generating a heterogeneous mix of cell sizes and shapes, leading to colony formation and growth as mentioned earlier (Fig. 3A, left).

Rojas *et al*. (2018) recently showed that the Gram-negative OM contributes substantially to the mechanical stability of the cell. This could explain the survival of *S. moniliformis* L-forms without extra osmolytes, at least in the presence of serum, and as recently described for *E*. *coli* L-forms in urine (Mickiewicz *et al*. 2019) or under low osmotic conditions (Osawa and Erickson 2019).

### OM growth associates with scission of cytoplasmic compartment in L-forms

**Figure 4.**
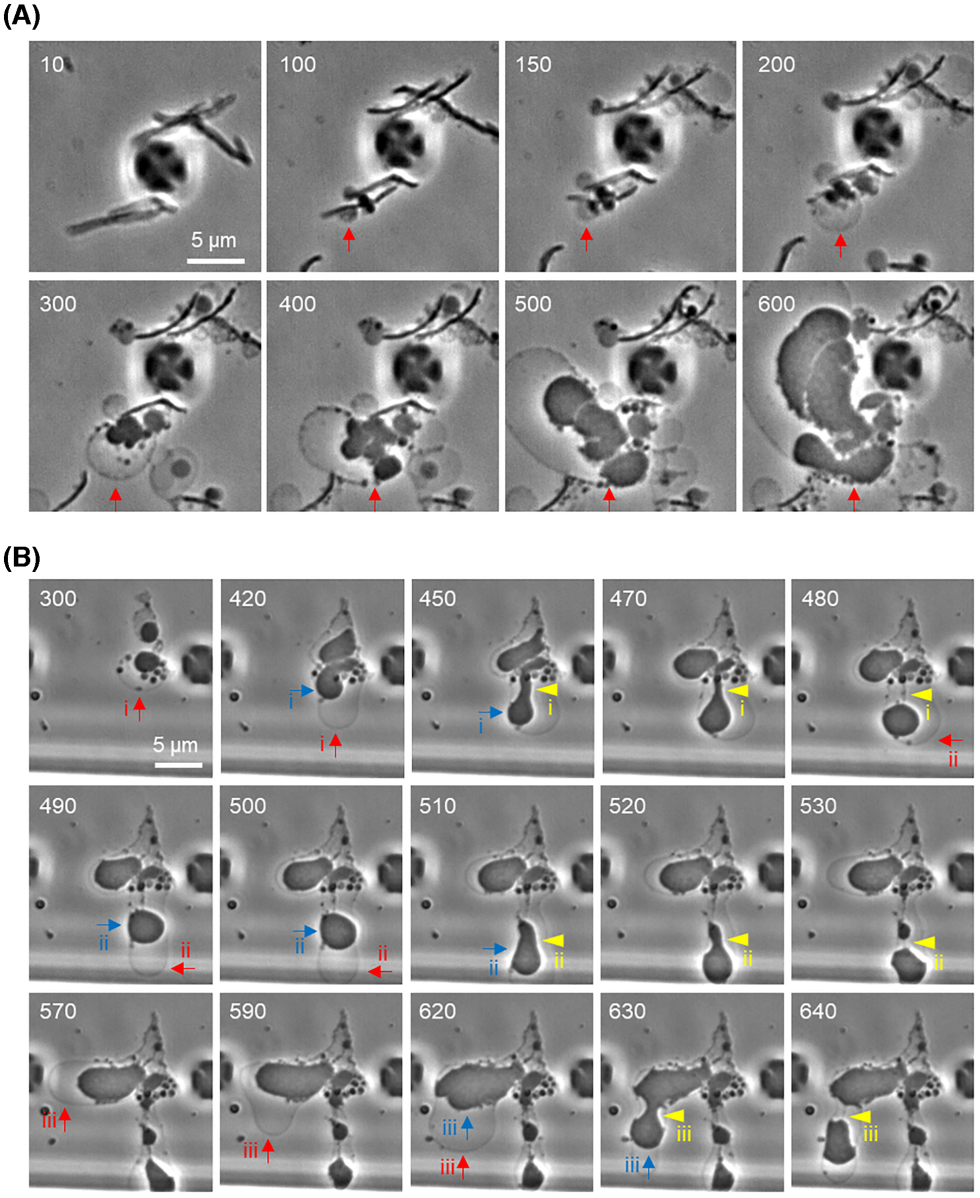
Membrane growth of *S. moniliformis* L-forms in a liquid medium. **(A)***Streptobacillus moniliformis* cells in NB with serum were treated with 200 μg/ml ampicillin in the CellASIC ONIX microfluidic system for time-lapse microscopy. Individual frames are extracted from Video S4 (Supporting Information). Numbers in the top left corner of each frame represent time (min) elapsed in the video. Arrowheads represent spontaneous formation of L-form-like structures. Arrow represents OM expansion followed by growth and deformations of L-form cell. **(B)** Phase-contrast micrographs of *S. moniliformis* L-forms during time-lapse experiments. Individual frames are extracted from Video S5 (Supporting Information). Numbers in the top left corner of each frame represent time (min) elapsed in the video. Red arrows show expansion of OM. Blue arrows show growth and deformation of cytoplasmic compartment. Yellow arrowheads show scission events.

L-forms use unusual blebbing, tubulation, vesiculation or budding mechanisms to proliferate (Kandler and Kandler 1954; Leaver *et al*. 2009; Mercier, Kawai and Errington 2013; Studer *et al*. 2016; Kawai, Mickiewicz and Errington 2018; Ramijan *et al*. 2018; Chikada *et al*. 2021). In several bacteria, L-form proliferation has been shown to be completely independent of the normally essential FtsZ-based bacterial division machinery or cell wall synthetic enzymes (Leaver *et al*. 2009; Mercier, Kawai and Errington 2014; Studer *et al*. 2016). Instead, at least in *B. subtilis*, L-form proliferation is brought about by an imbalance between cell membrane and volume growth, which drives membrane deformations leading to spontaneous scission (Mercier, Dominguez-Cuevas and Errington 2012; Mercier, Kawai and Errington 2013). However, in the time-lapse experiment described earlier for L-form switching and growth, it was difficult to follow the membrane dynamics due to the *S. moniliformis* L-forms being embedded in the agar. Although L-form growth is often less effective in liquid media (for unknown reasons; Mercier, Kawai and Errington 2014), we found that we could obtain useful time-lapse imaging in liquid medium with a microfluidic device as recently observed in *E*. *coli* L-forms (Chikada *et al*. 2021). Video S4 (Supporting Information) and Fig. 4A show a typical example of cell behaviour after ampicillin treatment in liquid medium (red arrow). First, large membrane blebs emerged that were phase pale, indicating that they were probably generated from OM expansion and an expanded ‘periplasmic’ space. The blebs then became occupied by multiple vesicles of a range of sizes, which were phase dark and therefore probably cytoplasmic. Some of these vesicles then enlarged, still within the enveloping OM, and took on the large irregular bloated appearance typical of L-forms.

Video S5 (Supporting Information) and Fig. 4B show an example of two L-form cells induced by ampicillin treatment. Initially, the OM of one of the L-form cells started to grow, generating an expanded periplasmic space (red arrow, i), and then the cytoplasmic compartment also grew and deformed (blue arrow, i), followed by scission events (yellow arrowheads, i). The OM continued to grow (red arrow, ii) and this was again followed by growth and deformation of the cytoplasmic compartment (blue arrow, ii), leading to scission (yellow arrowheads, ii). A similar series of steps was observed in another L-form cell (Fig. 4B, 570–640 min, iii).

Although this result suggested a critical importance of OM growth for proliferation of *S. moniliformis* L-forms, which was also recently described for *E. coli* L-forms (Osawa and Erickson 2019; Chikada *et al*. 2021), separation of progeny cells through OM scission was not evident under the conditions of our time-lapse experiments with liquid medium. This may reflect a physiological barrier to the proliferation of L-forms in liquid media. Given that significant OM expansion was not evident in the nonproliferative L-form-like structures or spheroplasts induced by sucrose (Table 1), excess osmolytes may prevent L-form growth by affecting OM expansion.

**Table 1.** Measurement of OM expansion.

	Area of OM (μm^2^)					
	L-forms^a^ (ampicillin)			L-form-like cells^b^ (sucrose)		
Time (min)	Cell 1	Cell 2	Cell 3	Cell 1	Cell 2	Cell 3
0	0.114	0.093	0.11	0.089	0.068	0.047
10	0.118	0.101	0.118	0.089	0.072	0.047
20	0.123	0.101	0.123	0.089	0.068	0.047
30	0.127	0.11	0.127	0.089	0.068	0.047
40	0.131	0.11	0.127	0.089	0.072	0.047
50	0.135	0.114	0.131	0.089	0.068	0.047
60	0.14	0.118	0.14	0.089	0.068	0.047
70	0.14	0.123	0.144	0.089	0.072	0.042
80	0.148	0.127	0.152	0.089	0.072	0.047
90	0.148	0.131	0.152	0.089	0.072	0.047
Increase over 90 min (%)	29.8	40.8	38.2	0	5.8	0

a L-forms were selected from a time-lapse experiment with ampicillin treatment used for Video S4 (Supporting Information).

b L-form-like cells were selected from a time-lapse experiment with sucrose treatment used for Video S2 (Supporting Information).

It has been shown that blocking the cell wall pathway leads to toxic ROS production via aerobic respiration (Kawai *et al*. 2015, 2019). Thus, reduction of RC activity or depletion of oxygen can promote robust L-form growth (Dienes and Weinberger 1951; Huber and Brinkley 1977; Kawai *et al*. 2015, 2019; Chikada *et al*. 2021). The persistent growth of a Gram-positive *E. faecium* (Kawai *et al*. 2019) and a Gram-negative *S. moniliformis* (which naturally lack a RC) during antibiotic treatment under aerobic conditions suggests that oxidative damage is a common impediment to the growth of L-form bacteria.

Recent work has rekindled interest in L-form field, largely based on the finding that L-form proliferation differs remarkably from that of walled cells. L-forms generate progeny cells by a range of poorly regulated shape perturbations. This work suggests that an imbalance between OM and cytoplasmic volume growth may act to drive L-form deformations leading to spontaneous scission in *S. moniliformis*. It seems likely that OM growth may be an important general factor underpinning the growth and proliferation of L-forms from diderm bacteria.

## Supplementary Material

fnab156_Supplemental_File

## References

[bib1] Allan EJ , Hoischen C, Gumpert J. Bacterial L-forms. Adv Appl Microbiol. 2009;68:1–39. 19426852 10.1016/S0065-2164(09)01201-5

[bib2] Chikada T , Kanai T, Hayashi M et al. Direct observation of conversion from walled cells to wall-deficient L-form and vice versa in *Escherichia coli* indicates the essentiality of the outer membrane for proliferation of L-form cells. Front Microbiol. 2021;12:645965. 33776978 10.3389/fmicb.2021.645965 PMC7991099

[bib3] Dienes L , Weinberger HJ. The L forms of bacteria. Bacteriol Rev. 1951;15:245–88. 14904355 10.1128/br.15.4.245-288.1951 PMC180721

[bib4] Domingue GJ , Woody HB. Bacterial persistence and expression of disease. Clin Microbiol Rev. 1997;10:320–44. 9105757 10.1128/cmr.10.2.320 PMC172922

[bib5] Elliott SP . Rat bite fever and *Streptobacillus moniliformis*. Clin Microbiol Rev. 2007;20:13–22. 17223620 10.1128/CMR.00016-06 PMC1797630

[bib7] Errington J , Mickiewicz K, Kawai Y et al. L-form bacteria, chronic diseases and the origins of life. Philos Trans R Soc Lond B Biol Sci. 2016;371:20150494. 27672147 10.1098/rstb.2015.0494 PMC5052740

[bib6] Errington J . Spotlight on… Jeff Errington. FEMS Microbiol Lett. 2017;364:fnx099. 10.1093/femsle/fnx099 28510651

[bib8] Freundt EA . Experimental investigations into the pathogenicity of the L-phase variant of *Streptobacillus moniliformis*. Acta Pathol Microbiol Scand. 1956;38:246–58. 13326444 10.1111/j.1699-0463.1956.tb03172.x

[bib9] Glaser P , Sharpe ME, Raether B et al. Dynamic, mitotic-like behavior of a bacterial protein required for accurate chromosome partitioning. Genes Dev. 1997;11:1160–8. 9159397 10.1101/gad.11.9.1160

[bib10] Glover WA , Yang Y, Zhang Y. Insights into the molecular basis of L-form formation and survival in *Escherichia coli*. PLoS One. 2009;4:e7316. 19806199 10.1371/journal.pone.0007316 PMC2752164

[bib11] Han J , He L, Shi W et al. Glycerol uptake is important for L-form formation and persistence in *Staphylococcus aureus*. PLoS One. 2014;9:e108325. 25251561 10.1371/journal.pone.0108325 PMC4177120

[bib12] Huber TW , Brinkley AW. Growth of cell wall-defective variants of *Escherichia coli*: comparison of aerobic and anaerobic induction frequencies. J Clin Microbiol. 1977;6:166–71. 330562 10.1128/jcm.6.2.166-171.1977 PMC274726

[bib13] Kandler G , Kandler O. Studies on morphology and multiplication of pleuropneumonia-like organisms and on bacterial L-phase, I. Light microscopy. Arch Mikrobiol. 1954;21:178–201. 14350641

[bib14] Kawai Y , Mercier R, Errington J. Bacterial cell morphogenesis does not require a preexisting template structure. Curr Biol. 2014;24:863–7. 24704074 10.1016/j.cub.2014.02.053 PMC3989771

[bib15] Kawai Y , Mercier R, Mickiewicz K et al. Crucial role for central carbon metabolism in the bacterial L-form switch and killing by β-lactam antibiotics. Nat Microbiol. 2019;4:1716–26. 31285586 10.1038/s41564-019-0497-3 PMC6755032

[bib16] Kawai Y , Mercier R, Wu LJ et al. Cell growth of wall-free L-form bacteria is limited by oxidative damage. Curr Biol. 2015;25:1613–8. 26051891 10.1016/j.cub.2015.04.031 PMC4510147

[bib17] Kawai Y , Mickiewicz K, Errington J. Lysozyme counteracts β-lactam antibiotics by promoting the emergence of L-form bacteria. Cell. 2018;172:1038–49. 29456081 10.1016/j.cell.2018.01.021 PMC5847170

[bib19] Klieneberger E . Origin, development and significance of L-forms in bacterial cultures. J Gen Microbiol. 1949;3:434–43. 18147756 10.1099/00221287-3-3-434

[bib18] Klieneberger E . The natural occurrence of pleuropneumonia-like organisms in apparent symbiosis with *Streptobacillus moniliformis* and other bacteria. J Pathol Bacteriol. 1935;40:93–105.

[bib20] Leaver M , Dominguez-Cuevas P, Coxhead JM et al. Life without a wall or division machine in *Bacillus subtilis*. Nature. 2009;457:849–53. 19212404 10.1038/nature07742

[bib21] Levaditi C , Nicolau S, Poincloux P. Sur le role étiologique de *Streptobacillus moniliformis* (nov. spec.) dans l’érythéme polymorphe aigu septicémique. C R Acad Sci. 1925;180:1188–90.

[bib22] Lovering AL , Safadi SS, Strynadka NC. Structural perspective of peptidoglycan biosynthesis and assembly. Annu Rev Biochem. 2012;81:451–78. 22663080 10.1146/annurev-biochem-061809-112742

[bib23] Markova ND . Eubiotic vs. dysbiotic human blood microbiota: the phenomenon of cell wall deficiency and disease-trigger potential of bacterial and fungal L-forms. Discov Med. 2020;29:17–26. 32598861

[bib24] Mercier R , Dominguez-Cuevas P, Errington J. Crucial role for membrane fluidity in proliferation of primitive cells. Cell Rep. 2012;1:417–23. 22832271 10.1016/j.celrep.2012.03.008

[bib25] Mercier R , Kawai Y, Errington J. Excess membrane synthesis drives a primitive mode of cell proliferation. Cell. 2013;152:997–1007. 23452849 10.1016/j.cell.2013.01.043

[bib26] Mercier R , Kawai Y, Errington J. General principles for the formation and proliferation of a wall-free (L-form) state in bacteria. eLife. 2014;3:e04629. 10.7554/eLife.04629 PMC4244569 25358088

[bib27] Mickiewicz KM , Kawai Y, Drage L et al. Possible role of L-form switching in recurrent urinary tract infection. Nat Commun. 2019;10:4379. 31558767 10.1038/s41467-019-12359-3 PMC6763468

[bib28] Nolan M , Gronow S, Lapidus A et al. Complete genome sequence of *Streptobacillus moniliformis* type strain (9901^T^). Stand Genomic Sci. 2009;1:300–7. 21304670 10.4056/sigs.48727 PMC3035246

[bib29] Onwuamaegbu ME , Belcher RA, Soare C. Cell wall-deficient bacteria as a cause of infections: a review of the clinical significance. J Int Med Res. 2005;33:1–20. 15651712 10.1177/147323000503300101

[bib30] Osawa M , Erickson HP. L form bacteria growth in low-osmolality medium. Microbiology (Reading). 2019;165:842–51. 30958258 10.1099/mic.0.000799 PMC7008213

[bib31] Ramijan K , Ultee E, Willemse J et al. Stress-induced formation of cell wall-deficient cells in filamentous actinomycetes. Nat Commun. 2018;9:5164. 30514921 10.1038/s41467-018-07560-9 PMC6279842

[bib32] Rojas ER , Billings G, Odermatt PD et al. The outer membrane is an essential load-bearing element in Gram-negative bacteria. Nature. 2018;559:617–21. 30022160 10.1038/s41586-018-0344-3 PMC6089221

[bib33] Schindelin J , Arganda-Carreras I, Frise E et al. Fiji: an open-source platform for biological-image analysis. Nat Methods. 2012;9:676–82. 22743772 10.1038/nmeth.2019 PMC3855844

[bib34] Silver LL . Fosfomycin: mechanism and resistance. Cold Spring Harb Perspect Med. 2017;7:a025262. 28062557 10.1101/cshperspect.a025262 PMC5287057

[bib35] Studer P , Staubli T, Wieser N et al. Proliferation of *Listeria monocytogenes* L-form cells by formation of internal and external vesicles. Nat Commun. 2016;7:13631. 27876798 10.1038/ncomms13631 PMC5123018

[bib36] Wu LJ , Lee S, Park S et al. Geometric principles underlying the proliferation of a model cell system. Nat Commun. 2020;11:4149. 32811832 10.1038/s41467-020-17988-7 PMC7434903

